# Movement Activity Based Classification of Animal Behaviour with an Application to Data from Cheetah *(Acinonyx jubatus)*

**DOI:** 10.1371/journal.pone.0049120

**Published:** 2012-11-19

**Authors:** Steffen Grünewälder, Femke Broekhuis, David Whyte Macdonald, Alan Martin Wilson, John Weldon McNutt, John Shawe-Taylor, Stephen Hailes

**Affiliations:** 1 Computational Statistics and Machine Learning, University College London, London, United Kingdom; 2 Department of Computer Science, University College London, London, United Kingdom; 3 Botswana Predator Conservation Trust, Maun, Botswana; 4 Wildlife Conservation Research Unit (WildCRU), Department of Zoology, University of Oxford, Recanati-Kaplan Centre, Oxford, United Kingdom; 5 Structure and Motion Laboratory, The Royal Veterinary College, University of London, London, United Kingdom; Tulane University Medical School, United States of America

## Abstract

We propose a new method, based on machine learning techniques, for the analysis of a combination of continuous data from dataloggers and a sampling of contemporaneous behaviour observations. This data combination provides an opportunity for biologists to study behaviour at a previously unknown level of detail and accuracy; however, continuously recorded data are of little use unless the resulting large volumes of raw data can be reliably translated into actual behaviour. We address this problem by applying a Support Vector Machine and a Hidden-Markov Model that allows us to classify an animal's behaviour using a small set of field observations to calibrate continuously recorded activity data. Such classified data can be applied quantitatively to the behaviour of animals over extended periods and at times during which observation is difficult or impossible. We demonstrate the usefulness of the method by applying it to data from six cheetah *(Acinonyx jubatus)* in the Okavango Delta, Botswana. Cumulative activity data scores were recorded every five minutes by accelerometers embedded in GPS radio-collars for around one year on average. Direct behaviour sampling of each of the six cheetah were collected in the field for comparatively short periods. Using this approach we are able to classify each five minute activity score into a set of three key behaviour (feeding, mobile and stationary), creating a continuous behavioural sequence for the entire period for which the collars were deployed. Evaluation of our classifier with cross-validation shows the accuracy to be 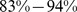, but that the accuracy for individual classes is reduced with decreasing sample size of direct observations. We demonstrate how these processed data can be used to study behaviour identifying seasonal and gender differences in daily activity and feeding times. Results given here are unlike any that could be obtained using traditional approaches in both accuracy and detail.

## Introduction

Advances in technology are allowing biologists to collect large amounts of high resolution data without the need to be physically present. This has the potential to give researchers a unique insight into aspects of animal behaviour and ecology that have, in the past, been more restricted due to environmental conditions and animal behaviour. Despite the easy availability of this technology, the uses of these data are still limited. For example, accelerometers, quantifying animal movement, allow us to investigate behavioural patterns and states through changes in activity. However, to date, most studies have used simple data-oriented methods of analysis: for example, threshold-based detection of active/inactive states [1] or classification into slightly richer behavioural states [2]–[4]. These techniques have often been used in marine systems where the animals in question are near to impossible to follow and observe [4]–[6]. Despite the fact that these techniques have given a valuable insight into behaviours such as underwater foraging in free-living penguins [7] and swimming and diving in marine mammals and birds [4], [5], it is hard to draw robust conclusions about actual/true behaviour from such analysis.

By combining the data from the dataloggers with behavioural observations from the field we have a powerful tool with which to investigate animal behaviour that is based on actual behaviour rather than purely on activity values and arbitrary thresholds. In a way, this would be difficult to achieve for most marine animals, but it is cetrainly feasible in terrestrial systems where animals are easier to observe. There has been an attempt to do this on domesticated and captive animals [8], [9] but, to date, this has never been done on wild animals. Generally, collecting large amounts of behavioural data on wild animals is extremely time consuming. By combining technology with field observations, large amounts of behavioural data can be collected in a relatively short space of time. With these data we can start to address more detailed behavioural questions, which is extremely valuable especially for elusive, far-ranging species that cannot be monitored continuously and do not occur in high densities.

The main difficulty that needs to be overcome with this approach is the processing of the data. The amount of data is typically far too large to be processed by hand; moreover, it is a priori unclear that recorded activity measurements are sufficient to predict actual animal behaviour. In this paper, we study this question on animals that are equipped with tracking collars. These collars typically comprise a VHF (very high frequency) beacon and/or a GPS (global positioning system) unit to provide locations of an animal in its environment. Furthermore, collars are often equipped with an accelerometer that senses acceleration on one or more axes. In the medical world, for example, accelerometers are used to sense movement and estimate energy expenditure and create simple activity diaries [10]. The processing of such signals can range from simple estimates of peak value or magnitude over time or average value which provides information about orientation. Here, we examine data from a commercial Vectronics collar, which combines two-dimensional accelerometer data over a five minute period to form a value between 0 and 255; little additional information is available about the nature of the algorithm used for data reduction. We are interested in establishing whether such a simple signal can be used to differentiate between different behaviours.

We address this challenge with machine learning techniques. Our method is based on a Support Vector Machine (SVM, [11]) which is a popular and powerful method for classification. As the SVM does not consider temporal information, we post-processed the SVM results with a Hidden Markov Model (HMM) to introduce dependencies over time. This method maps the recorded activity into a sequence of behaviours that can be post-processed to address scientific questions on animal behaviour. We also illustrate the fact that only a relatively small number of behavioural observations are required for a robust analysis, ideal for animals that are difficult to follow.

To validate the approach, and to show its potential, we applied the method to an activity dataset from six free-ranging cheetah (*Acinonyx jubatus*). With fewer than 10,000 individuals left in the wild, cheetah are rare. The fact that they are fairly elusive carnivores, have large home-ranges and are often solitary makes them difficult to locate and, therefore, makes it impossible to obtain large amounts of behavioural data in a short period of time. Our technique allows us to extrapolate behavioural data for several individuals simultaneously and then thoroughly to investigate the different behavioural states. We demonstrate the usefulness by addressing questions like: how often do cheetah feed? For how long do they feed? How regularly do they feed? When, during the day, are they active? How do certain variables like season affect feeding or activity?

## Materials and Methods

### Ethics Statement

In compliance with Botswana law, all immobilisation and deployment of radio-collars were carried out by a Botswana-registered veterinarian. Cheetahs were immobilised according to protocol by [12], and sedation time was kept to a minimum, usually no longer than 1 hour. All animal handling protocols conformed to the standards of the American Society of Mammalogists [13] and were approved by both the Zoology Ethical Review Committee, a subsidiary of Oxford Universities Animal Care and Ethical Review (ACER) Committee (License CER-FB2008) and by the Botswana Department of Wildlife and National Parks (permit EWT 8/36/4). All cheetahs recovered following immobilisation and showed no signs of distress. On completion of the study, all collars were removed.

### The Classifier

We use a Support Vector Machine (SVM) in combination with a hidden Markov approach for the classification of the data. We train the SVM to predict three different classes: stationary, mobile and feeding. We apply the SVM independently to each five minute activity value. Applying the classifier to the full activity sequence gives us a sequence of predicted behaviour. The classifier, however, does not take temporal information into account. This sometimes leads to “obvious” misclassifications like: we observe a long sequence of stationary behaviour (e.g. sleeping) surrounding a single feeding data point. Such misclassifications can be eliminated by incorporating an understanding of the likely temporal behaviour of the animals. We included temporal information by “smoothing” the behaviour sequence with the help of a hidden Markov approach. In the following, we give some background on the SVM and the hidden Markov approach, and we discuss the extent to which we can validate the predicted behaviour sequence.

#### Support Vector Machine

The SVM is a popular method for learning classifiers as it is robust and fast [11]. In the following, we explain the central concept of the SVM to illustrate the operation of the method. In general, a classification problem consists of a set of input vectors 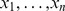 and a set of corresponding labels 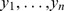. In the easiest case, there are two classes and the labels are binary 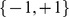. For simplicity, assume that our inputs are 2-dimensional and that we can separate the two classes in the input space with the help of lines (i.e. there exists a line such that all the positive examples are on one side and all the negative examples on the other). Usually, many different lines are able to separate the two classes. So the question is which of these lines make good choices. The SVM is a so-called large margin method as it finds the specific line that maximizes the margin from the line to the two classes. That is, it maximally separates the two classes. Intuitively this is a good choice as the classifier is robust against new data points that lie slightly outside of the observed class boundaries. Beside this intuition there are formal guarantees for the performance of an SVM [11] and the SVM has been shown to be very robust in various applications.

In most cases, lines are insufficient to separate classes. SVMs rely on the so-called *kernel-trick* to increase the separation between classes. The kernel-trick makes use of a kernel 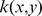 that measures similarities between elements 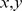 and needs to fulfill certain properties to be applicable (it must be positive semi-definite). A frequently used kernel is the Gaussian kernel:
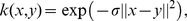
where 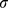 is a hyper-parameter and 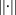 the Euclidean norm. Using the Gaussian kernel implies that the data is embedded in an infinite dimensional space and all operations are applied in this space.

In our application, the input elements were 2 dimensional vectors representing aggregated 2D accelerometer readings (see the Data Collection section on p. 6 for details), and we had three classes of behaviour: stationary, mobile and feeding. We trained three classifiers, one for each class: the positive examples were the elements 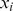 with the correct behaviour and negative examples were the elements with one of the remaining two behaviours. We used the SVM package from [14] for our experiments and we used the Gaussian kernel. We used all available labeled data for one cheetah for training and applied it to the whole set of activity measurements of that cheetah (the number of training data points and the total data size is shown in Table 1).

**Table 1:** Classification performance.

Nr.	Individual	M1	M2	F1	F2	F3	F4
1	Percent correct (overall)	94.03%	91.54%	90.87%	94.24%	83.75%	90.16%
2	Percent correct (feeding)	93.00%	52.17%	94.83%	100%	22.58%	65.96%
3	Percent correct (mobile)	96.97%	86.84%	73.77%	90.72%	75.31%	68.67%
4	Percent correct (stationary)	92.81%	99.63%	97.54%	94.96%	98.83%	99.37%
5	False positives (feeding)	2.00%	2.17%	5.17%	0%	6.45%	0%
6	False positives (mobile)	9.09%	23.68%	0%	7.22%	20.99%	2.41%
7	False positives (stationary)	6.54%	5.22%	15.57%	6.47%	15.79%	13.25%
8	Labeled data points	352	390	241	278	283	447
9	Feeding data points	100	46	58	42	31	47
10	Mobile data points	99	76	61	97	81	83
11	Stationary data points	153	268	122	139	171	317
12	Total data points	114302	94928	100233	78434	79865	106013

The table shows the cross-validation performance of our method on the labeled data points. The method is used to classify all data points. Row number 1 shows the overall accuracy. Rows 2–4 show the individual accuracy of detecting the three different classes correctly. Rows 5–7 show the false positive rates, that is the probability that a data point is labeled to belong to class X while it is actually not class X. The number of labeled data points are shown in row 8–11 and line 12 shows the number of data points that we classified with the trained method.

#### Hidden Markov approach

We smoothed the classification sequence with the help of a hidden Markov approach. This requires that the SVM output includes a probability value for how certain the predicted class is. A standard approach for obtaining this is to apply the logistic function to the distance from the hyperplane [15]. The logistic function is.
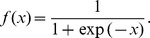



The value of 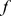 is between 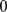 and 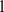 and can thus be interpreted as a probability. We applied the logistic function to the three trained classifiers which resulted in three sequences  and 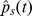. From these, we generated probabilities that the behaviour is feeding at time 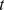 (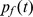), mobile (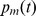) or stationary (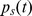) given the observation 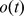 in the following way:

1. The animal can only show one behaviour at time 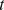 and hence two of the behaviours should not be present. This leads us to use:
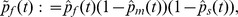


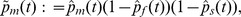


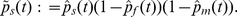



2. We normalised the values from the first step such that they sum to unity for each time step 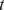, i.e.
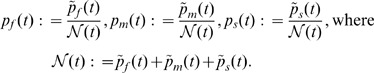
(1)


We constructed a hidden Markov model (HMM, [16]) with three states (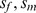 and 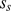) that encode the three behaviour classes. We interpreted the three sequences 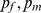 and 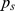 as the conditional probabilities 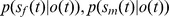 and 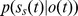 that the behaviour is feeding, mobile or stationary at time 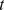 given the observation 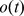. For our algorithm we need to calculate the likelihoods 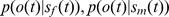 and 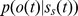. These can be computed with the help of Bayes rule: 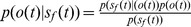 etc. If prior knowledge about the the probabilities for certain observations or the probabilities for certain states is known then it can be exploited here. In our experiments, we had no prior knowledge and we used uniform distributions for both 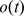 and 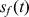 etc.

In addition to determining these likelihoods it is necessary to define a transition model for the states. For this, we used.
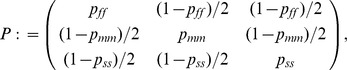
where 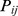 is the probability of transitioning from state i to state j with state 1 being feeding, state 2 mobile and state 3 stationary. Our motivation for this form is that we expect the current behaviour to continue with high probability (e.g. sleeping) and expect the behaviour to change with a lower probability. We used the same probabilities to switch to either of the two alternative behaviours (e.g. from stationary to mobile or feeding) to keep the number of parameters small. Our main interest in using this smoothing is to rule out single events like feeding in longer sequences of stationary behaviour. We expected that the smoothing should be relatively robust to the particular choice of values 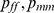 and 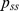, so long as they were sufficiently high. We used values of 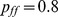 and 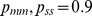 as feeding is likely to be shorter than stationary or mobile behaviour. We analyzed the effect of changing 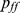 (robustness results can be found in the next section). Note that the choice of this value implies an expected length of time each state is maintained before a transition is made to a new state. The value of 0.8 corresponds to one hour twenty minutes, a value that agrees in magnitude with our observations of feeding. The value 0.9 corresponds to seven and a half hours and is at the upper end of what we would expect for stationary or mobile behaviour - refining the parameter up to the second decimal digit might lead to slight improvements, but we decided, in this case, that parameter tuning to this extent was a second order improvement.

We also need to specify initial probabilities for the three states. We used 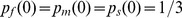.

Given 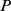 and the likelihoods 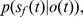 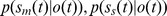 we wish to calculate the most probable state sequence. This sequence can be calculated sequentially with the Viterbi algorithm [16]: Let.
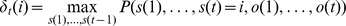
be the highest joint probability for any sequence of states 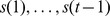 and (fixed) observations that visits state 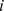 at time 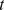.

The sequence can be calculated recursively as.
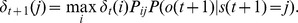



The initial value for the sequence of 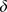
's is.
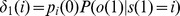
and the probabilities 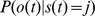 are the values from the SVM (eq. 1, p. 4).

Once we compute 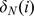 for N the final point of the sequence and 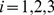, we select the final state as 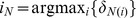. We now can identify the most probable sequence of states working from this last state backwards, with the state 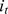 at time 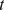 given by.
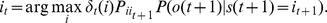



The state sequence output by the algorithm is 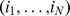.

#### Test-set performance and parameter robustness

We evaluated the performance of the method based on the recorded data and in terms of robustness of the parameters of the Markov chain. The performance of the method was assessed with the help of cross-validation: our training data consists of multiple time-delimited segments of observations of the cheetah, e.g. the cheetah was observed on Monday from 13∶00 to 15∶00 then again on Friday from 10∶00 to 11∶00 etc. We used each of these segments as a validation set and the remainder of the data as training data for the method. We then evaluated the performance on this validation set and we averaged the performance over all possible training-validation set combinations. The resulting percentage of correct classification is shown in Table 1.

**Figure 1:**
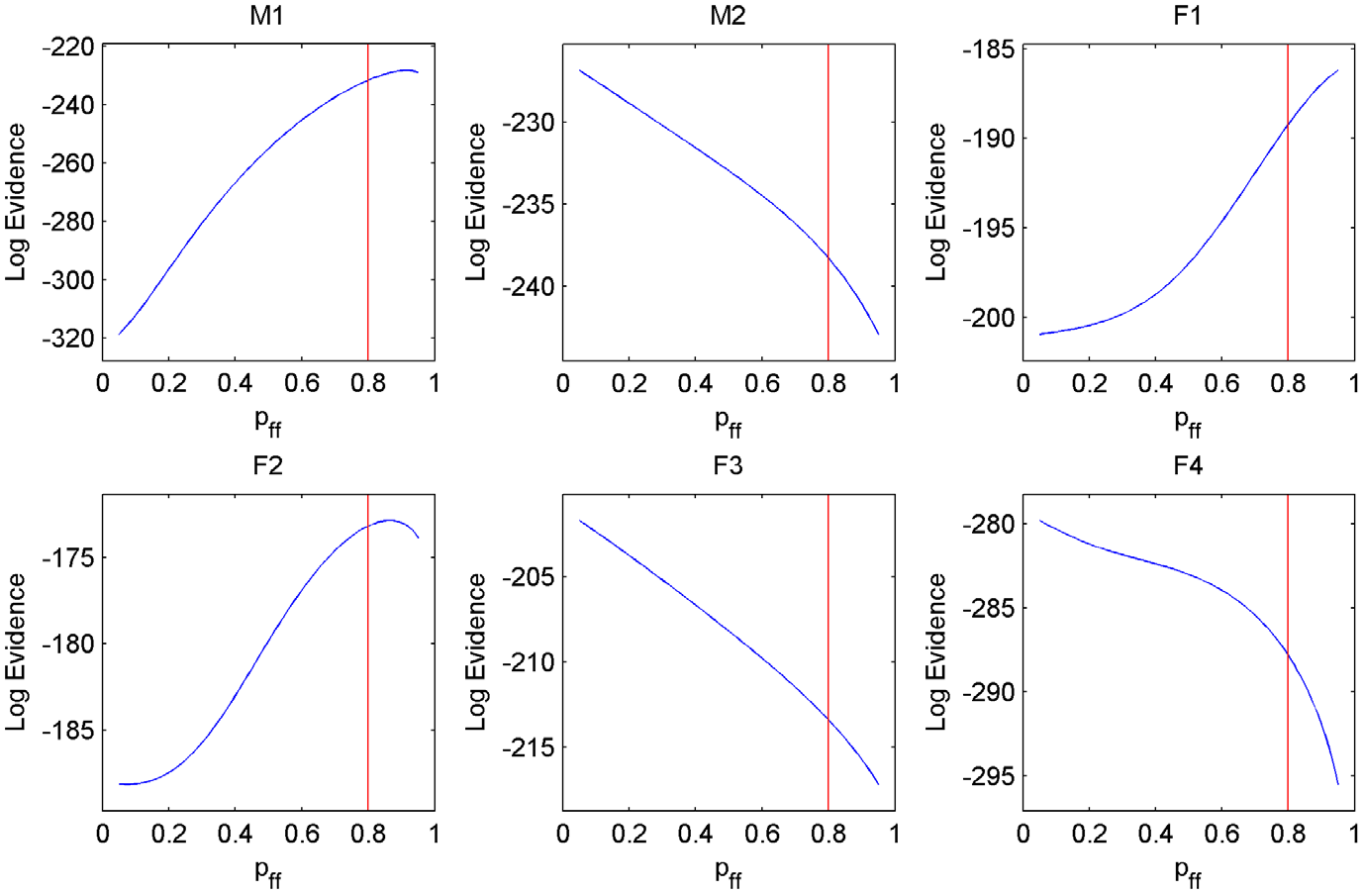
The figure shows how robust our method is to changes in the parameter 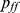. On the x-axis the parameter 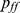 is varied and on the y-axis the corresponding evidence is plotted. The red(vertical) line marks the parameter that we used for the experiments in the rest of the paper.

Beside the performance we evaluated the sensitivity of the results to parameter changes in the Markov chain. For this we measured the log evidence over the same segments that we used for the cross validation. The evidence can be calculated by summing the likelihoods of all possible paths in the HMM. Like the likelihood, the evidence can be calculated efficiently through recursion:
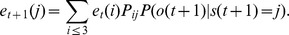



The initial value for the sequence of 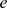 s is.
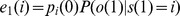
and the probabilities 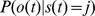 are again the values from the SVM (eq. 1, p. 4). Fig. 1 shows the results on our data.

### Data Set and Data Collection

#### Study area

The data for this study were collected in the Okavango Delta ecosystem, a permanent inland delta situated in Northern Botswana, Africa. The core study site (19°31′S, 23°37′E; elevation ca. 950m) encompasses an area of approximately 1 320 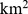, which includes the Southern part of the Moremi Game Reserve and the adjacent Wildlife Management Areas [17], [18]. The climate is characterised by two distinct seasons: the wet and the dry season. The wet season runs from the beginning of November to the end of March and the dry season runs from the beginning of April to the end of October.

#### Data collection

During the period from 2008 to 2011, six cheetah (four females and two males) were fitted with GPS (Global Positioning System) radio collars (Vectronics Aerospace GmbH, Germany). As required by law, a Botswana registered veterinarian was responsible for the immobilization procedures needed to fit the GPS radio collars. The cheetah were typically immobilised using a combination of Zoletil and Medetomidine [19].

The GPS radio-collars were embedded with bi-axial accelerometers that recorded both the forward-backwards movement and sideways movement every five minutes for the entire time that the collars were deployed. The collars were deployed for an average of 332 days (min: 276; max: 373) In addition to the activity measurements that were recorded by the collars, behavioural observations were collected in the field for each of the six cheetah independently. Behavioural observations were obtained by having each collared individual continuously followed by a researcher in vehicle for a minimum of one hour on any given day. During the periods of observation, three behavioural states were recorded: stationary, mobile and feeding. Each time the focal animal changed its behavioural state, the exact time was recorded using a handheld GPS device (Garmin eTrex HC; Garmin, Olathe, Kansas, USA). An average of 31 hours 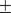 8 hours (mean 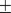 SEM; min: 21hr 59min; max: 40hr 49min) of observations were recorded during the study period. These behavioural observations were then synchronised with the activity data from the collars to give the labeled dataset. This resulted in an average of 332
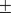
78 labeled data points per individual (mean
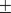
SEM; min: 241 data points; max: 447 data points). Of this labeled dataset, we only used labeled data points that were dominated by one discrete behaviour during the 5 minute interval, i.e. we excluded, for example, data points for which, in the 5 minutes, an animal was both feeding and mobile. The resulting number of labeled data points is shown in Table 1.

## Results

**Figure 2:**
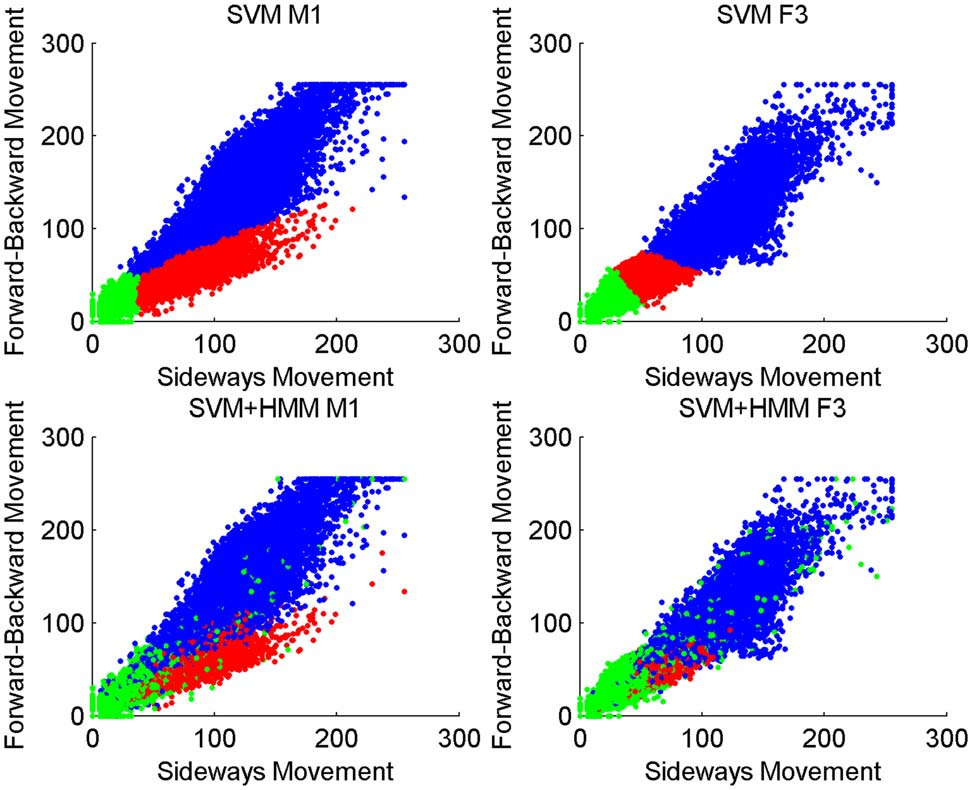
The figure shows, in the top row, all data points for M1 and F3 together with the class which has the highest probability based on the SVM. The bottom row shows all data points for the same individuals with the difference that the class is now assigned by the post-processed SVM classifier (SVM+HMM). The colour codes the class: green is stationary, dark blue is mobile and red is feeding.

We developed a SVM-based classifier that allows us to classify behaviour of animals over a long period of time based on activity measurements and a small set of labeled data. We applied the method to data from six cheetah. The method uses two types of data 1) the activity data from the data loggers deployed on each individual cheetah and 2) behavioural observations of cheetah in the field. These data were synchronised to produce a labeled data set. The labeled data were then used to train a SVM classifier. Fig. 2 shows trained classifiers for cheetah M1 and F3 in the top row.

The SVM output was smoothed with the help of a HMM over the complete set of activity measurements giving a detailed, 5 minute resolution, account of cheetah behaviour for the entire period for which the data loggers were deployed. The HMM has three hidden states, which encode feeding, mobile and stationary behaviour, and we interpreted the SVM output as the likelihood of the hidden states given an observation. Beside that, we used a transition matrix for the HMM states that has three parameters: 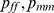 and 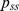 the probability of staying in the feeding, mobile or stationary state respectively. The resulting smoothed classifier for M1 and F3 is shown in the bottom row of Fig. 2. The technical details of the classifier, the collars and the data can be found in the methods section. In the next section we evaluate the performance of the method. Following that, we demonstrate the capabilities of the approach by applying the method to the cheetah data. We analyse gender differences for daily behaviour, feeding and inter-feeding times; we then analyze the effect of the seasons (dry and wet season). These preliminary analyses are presented to illustrate the capabilities of our method and the sort of questions that can be addressed.

### Evaluation

We evaluated our method in two different ways: 1) we estimated the classification performance with a leave-one-out cross validation; 2) we tested the dependency of the HMM performance on a critical parameter.

The cross validation results are shown in Table 1. The top row lists the performance over all three behaviour classes. The performance is a solid 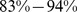 accuracy for classifying single data points correctly. The three rows below list class individual accuracy. Here, the performance is dependent on the number of observations we have for the different classes. In particular, one can observe that the feeding accuracy can be low if we have too few feeding observations (∼50).

The three rows below report the false positive numbers. Here, one can observe that the classes with few observations have low rates, e.g. when the classifier predicts feeding then there is a 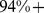 probability that the data point is really feeding.

The bottom line of the cross validation performance evaluation is that our method might miss single feeding or mobile data points for some of the individuals, but it will hardly ever miss whole sequences of behaviour that consist of multiple sequential data points. The probability for missing a whole sequence is 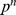, where 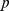 is the probability of an error and 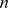 the length of the event. For example, F3 has the worst feeding performance, which results in an error rate of around 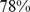 per data point. If feeding takes one hour then we have 12 data points and the chance of missing it is 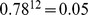 if we assume the observations are independent. In this worst case we have 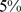 possibility of missing an average length feeding event completely.

Beside the cross-validation we tested the sensitivity of the classifier to the HMM parameter 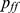. 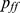 is the most interesting parameter of the three HMM parameters as we have few feeding observations for some individuals and because feeding times are in a range that could potentially be covered by a large portion of the parameter space. In detail, one expects feeding to lie in the range from 2–3 data points (that is 10–15 minutes; e.g. a small kill) up to 24–36 data points (2–3 hours; e.g. a big kill). This corresponds roughly to parameters 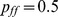 and 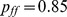. In contrast, for mobile and stationary we would expect parameters in the range of 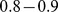.

We measured the sensitivity with the help of the Bayesian evidence for our model, that is the probability under our model that we observe certain observations. As in the cross-validation here we also used a leave-one-out approach: we trained the SVM on all but one set of data points, determined the evidence on the omitted set, and repeated that for each set. Fig. 1 shows the results. The higher the value is in the plot, the higher the evidence and the better the prediction performance is. The plots show two things: 1) for the three cheetah for which we have the lowest feeding prediction accuracy as a result of too few feeding observations (M2,F3,F4) we have evidence plots that peak at 0. So, the overall performance is maximised by ignoring feeding altogether; 2) for M1 and F2 we have optima between 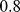 and 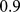 while F1 does not peak in the range we tried. In experiments, we used a single parameter 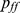 with a value of 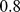 (the red lines) for all 6 cheetah to keep the number of parameters in the model small. Increasing 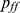 to a value slightly higher than 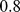 might improve the performance for M1,F2,F1 but will decrease the performance for the other three.

The conclusion of the experiment is that the evidence indicates whether there are too few observations or, in the case in which we have enough observations, it shows us which average feeding length is best for explaining the data and how sensitive the results are to this parameter.

### Application

#### Gender differences

We have data from two male and four female cheetah. We analysed the effect of gender on the daily behaviour, the feeding length and the time between feeding events.

**Figure 3:**
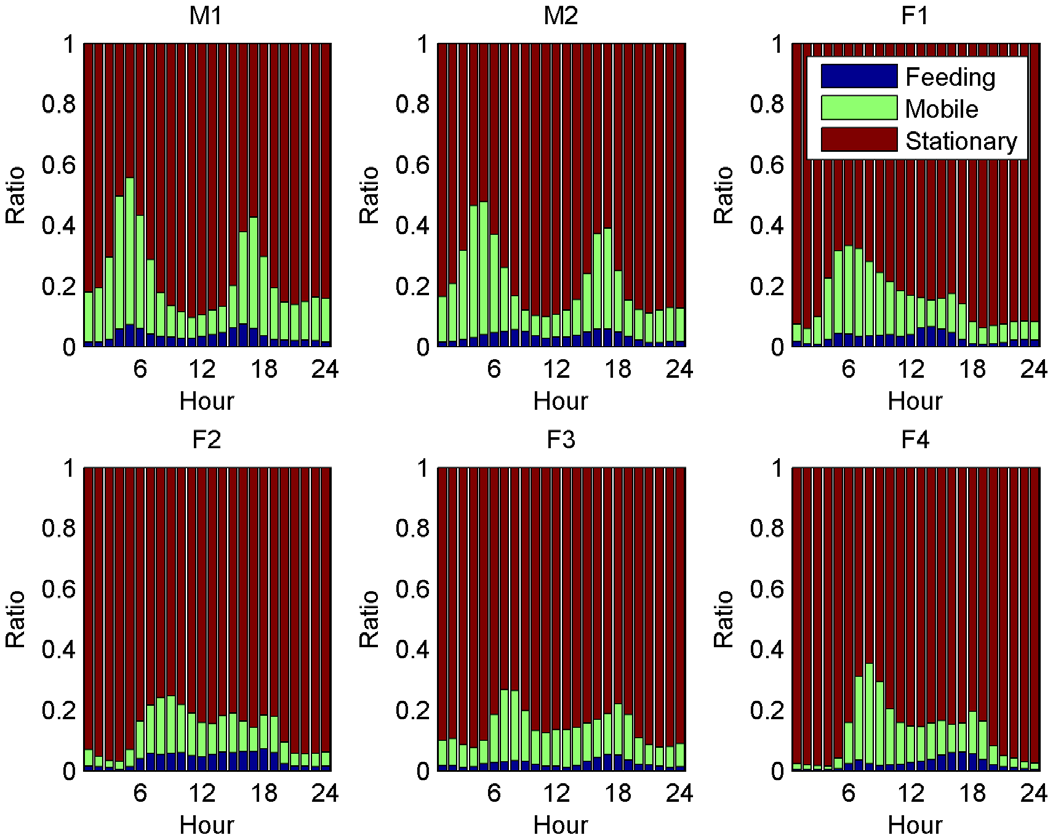
The classification of the daily activity of the six individuals is shown in the figure. The activity is colour coded with feeding being at the bottom, mobile in the middle and stationary at the top. The activity is classified based on the hour of the day (local time) and normalized to one. Sunrise during wet season is between 5∶19 and 6∶25 and during the dry season between 5∶30 and 7∶01. Sunset during the wet season is between 18∶16 and 19∶11 and during the dry season between 17∶35 and 18∶31.

Fig. 3 shows the daily behaviour for the six cheetah. The behaviour is split into three categories: feeding, mobile and stationary. Feeding is shown at the bottom, mobile in the middle and stationary at the top. The top left plots show the results for the males and the other four the results for the females.

All six cheetah have activity peaks at time of day around 6∶00 - 7∶00 and 17∶00 - 18∶00. The activity peaks for males are much higher than those for females. Similarly, the night activity for the males is considerably higher than the night activity of the four females. In general, the six cheetah are active for about 20% or less of the time.

Feeding occurs for all six cheetah mainly between 6∶00 and 19∶00 with some feeding events during the night. For both male and female cheetah there are slight peaks in feeding in the morning and evening. Male and female cheetah seem to have about the same number of night feeding events, even though males are far more active at night.

**Figure 4:**
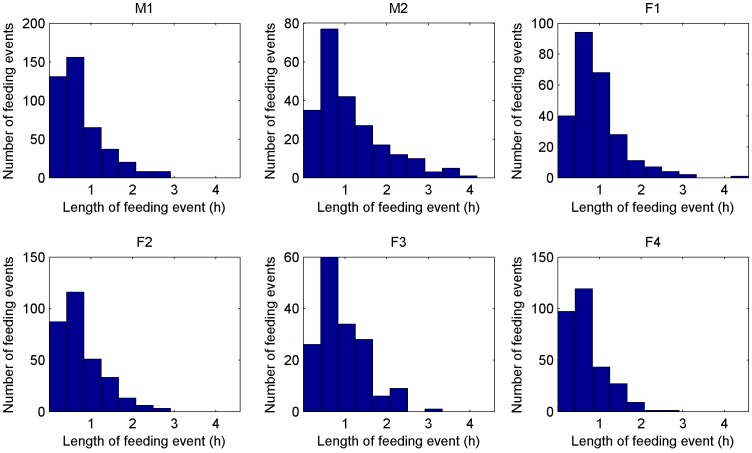
The figure shows histograms of the length of feeding events for the six individuals. On the x-axis the length is plotted and on the y-axis the number of events in the data with the respective length.

**Figure 5:**
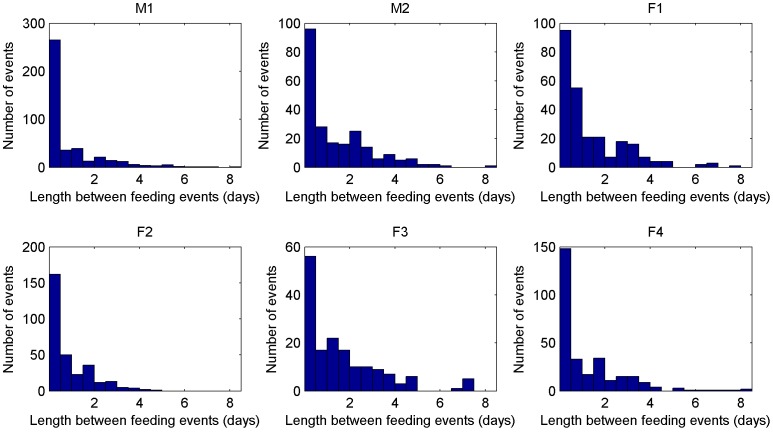
The figure shows histograms of the length between successive feeding events. On the x-axis the length is plotted and on the y-axis the number of events in the data with the respective length.

Fig. 4 shows the distribution over length of feeding and Fig. 5 shows the time between feeding events for the six individuals. The setup of the plots is the same as in Fig. 3: the results for the two males are shown in top left and the results for the four females are shown in the other plots.

The feeding length plots are similar for all six cheetah. On the left of each plot, short feeding events of around 20 minutes are shown. The rate for these events is relatively low. The highest rate of feeding events is around 20–40 minutes for all six cheetah. The rate for feeding events drops exponentially to the right of the peak and reaches zero between 3–4 hours.

The inter-feeding plots are similar. The 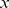-axis encodes days rather than hours. The highest peak is reached at the leftmost interval for all six cheetah. To the right of the peak, one seems again to have an exponential drop for all six cheetah (the scales vary for the six cheetah depending on the height of the peak on the left). In contrast to the feeding length plots there are “dips” in all six inter-feeding plots where the rate is considerably lower. The longest inter-feeding times are around 8 days for all six cheetah.

Table 2 summarises the rate of feeding. We calculated this rate by measuring the number of days that passed between feeding events. Based on this analysis, all six cheetah feed on average every second day.

**Table 2:** Feeding rate per day.

Individual	M1	M2	F1	F2	F3	F4
Feeding rate(overall)	0.5	0.43	0.46	0.58	0.43	0.45
Feeding rate(dry)	0.53	0.43	0.43	0.6	0.39	0.38
Feeding rate(wet)	0.46	0.43	0.49	0.58	0.46	0.54

The table shows how often each individual feeds on average per day.

#### Seasonal differences

In the second set of experiments, we studied effects of season on daily behaviour, feeding length and time between feedings. We show plots for three individuals. We selected the individuals for which we had the best feeding and mobile accuracy, that is M1, F1 and F2.

**Figure 6:**
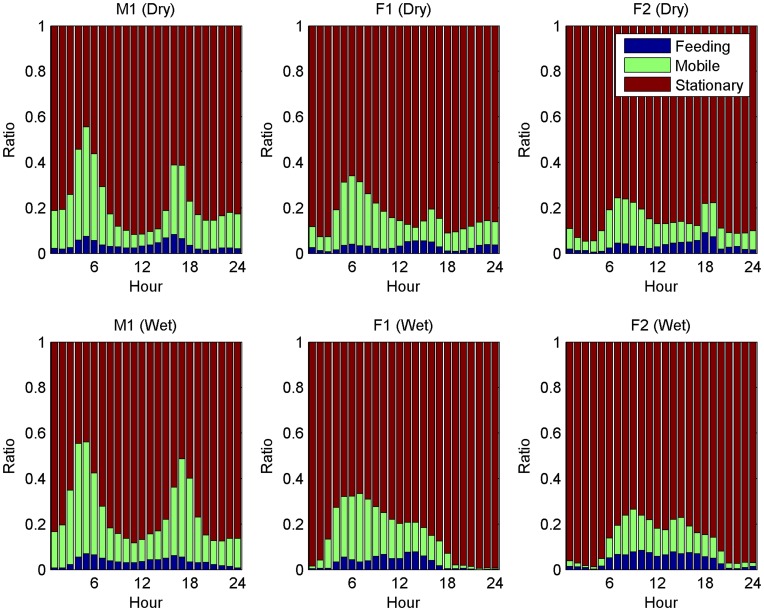
The figure shows effects of the season (dry/wet) on the daily activity of three individuals. The figure is similar to figure 3 with the main difference that the data is split into dry and wet season. The top row shows the activity of three individuals in the dry season and the bottom row the activity of these three individuals in the wet season.

Fig. 6 shows the difference in daily behaviour between wet and dry season for these three cheetah. The behaviour of M2 was very similar to M1. The plots shown for the two females are also similar to the omitted two.

One can observe that there is no obvious difference in the behaviour of the male cheetah between the wet and dry season. Compared to that, there are multiple obvious changes for the female cheetah: first, night activity decreases considerably during the wet season. The activity during the night in the dry season is considerable and is around half as high as the activity from M1. Also, there seems to be substantial amounts of feeding, especially for F1. The feeding rate of F1 during the night is, at its peak, nearly as high as that during the day. Second, during mid-day, the activity slightly increases and the feeding rate goes up.

**Figure 7:**
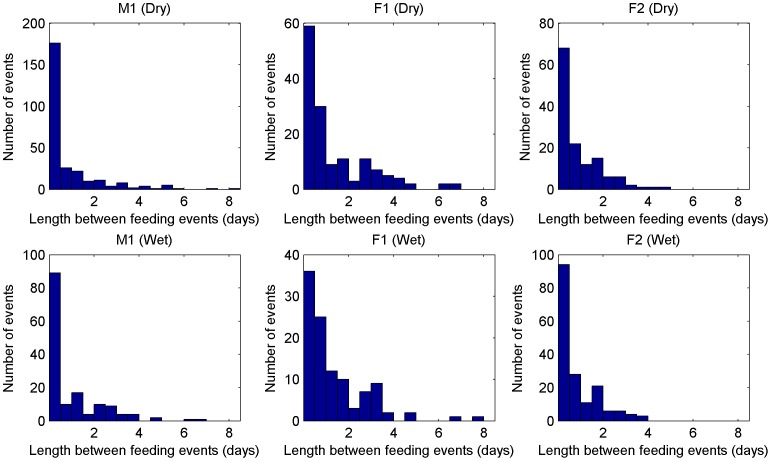
The figure shows effects of the season (dry/wet) on the length between feeding events of three individuals. The figure is similar to figure 5 with the main difference that the data is split into dry and wet season. The top row shows the length between feeding events of three individuals in the dry season and the bottom row the length between feeding events of these three individuals in the wet season.

We omitted a seasonal plot for the feeding length as there seems to be little difference between the dry and wet seasons. Fig. 7 shows the inter-feeding times for the three individuals. The seasonal differences are also not significant: for M1 the curve following the first peak is more smooth in the dry season, while in the wet season one has smaller peaks. For F1 there seems to be a slight increase of inter-feeding times to between 1–3 days. Finally, the F2 plots seem to be more or less equivalent for the wet and dry season.

Furthermore, Table 2 suggests that, for male cheetah, the feeding rate stays either constant with season or drops slightly in the wet season. In contrast, the feeding rate increases for 3 out of 4 female cheetah in the wet season.

## Discussion

Owing to recent advances in various technologies applied to the study of animal behaviour, biologists can now record high resolution behavioural data over extended periods. However, data from long, otherwise unobserved, periods are often insufficient to draw biologically relevant conclusions because meaningful information can be obscured within the vast quantities of accumulated dimensionless data. As a consequence, there is a temptation to make use of ad hoc analytical methods. We demonstrated in this work that generally applicable machine learning methods can be used to process and present such accumulations of data to identify specific behaviours. In particular, we applied a SVM together with a Hidden-Markov Model to classify activity reliably into three categories: stationary, mobile and feeding. Based on this classification sequence, we derive diagrams that can provide insights into cumulative behaviour deriving from continuous recording over periods that would be impossible by direct observation.

We evaluated our approach with the help of a cross-validation and evidence analysis. The accuracy obtained from these rely on the assumption that the data we used for training is representative of the data from the whole year. This will most likely be the case for large parts of the data as long as there is neither an injury nor damage to the collar. If there is sporadic behaviour that we do not record during the behavioural observations in the field then it it possible to get systematic misclassifications. For example, all four collared females had cubs and were lairing during part of the data collection. As we had no field observations of the nurturing behaviour of these cheetah, it is possible that some of this behaviour caused misclassification of other behavioural states. This effect is very typical for experimental studies as it is nearly always the case that we cannot observe animals in all situations.

Fig. 2 demonstrates that for our method it is crucial to have enough characteristic data for the different classes of behaviour. The left side shows the performance of the SVM classifier and the combined SVM+HMM classifier for M1. For M1 the classifier achieved high performance and one can observe that the HMM does not change the classifier region fundamentally but labels single data points differently compared to the pure SVM classifier. The right side shows the classifiers for F3 where the classifier achieved weaker performance. The feeding region based on the SVM classifier looks significantly different from the M1 plot. It can be expected that this is due to a lack of feeding observations. Finally, the HMM changes the shape of the feeding region significantly and makes it more similar to the M1 plot. However, it cannot fully account for the initial shortcomings of the SVM classifier.

An alternative to our approach of applying a HMM together with a SVM would be to use only a HMM by defining a suitable observation model and learning the HMM transition model with the help of the Baum-Welch algorithm [16]. A disadvantage of this approach is that it is not straightforward to make use of the labeled data, i.e. the behavioural observations. Furthermore, as our evidence analysis shows, the Baum-Welch algorithm would, most likely, suppress the feeding state altogether for three of the six cheetah and thus result in a model that could not be used to study feeding behaviour.

A further alternative would be to focus on the SVM side and drop the generative model approach that we pursue. This could be done with the help of structured output learning like in [20].

Given the recent development and advancement of technology one can expect that the amount of recorded data will continue to grow as hardware becomes cheaper and recording frequencies increase due to better batteries and less energy-consuming sensor devices. Furthermore, with new technology it might be possible that, in the near future, behavioural observations will be made remotely through recording devices like cameras.

Field studies are important either to verify and/or to raise new hypotheses about animal behaviour. It is important that the data and the data processing method allows to study or formulate hypotheses by providing insight into animal behaviour. We demonstrate this capability by raising a number of questions and hypotheses concerning the animal behaviour that merit further exploration.

The first question concerns the activity per day plot (fig. 3). The males show a high activity at night in contrast to the females (around twice the activity of females during the dry season). One possible reason for this is that male cheetah hunt at night. Cheetah hunts are very short and we cannot retrieve them from our 5 minute average and, hence, we cannot directly verify hunting behaviour. However, as the feeding rate is low at night, one can assume that only a small part of the activity is due to hunting. A likely alternative hypothesis is that since male cheetah are territorial, they patrol their territory at night whilst females are not territorial and therefore do not need to patrol [21], [22].

Another question concerns the “dips” in the histograms in Fig. 5. A likely explanation is here that the dips reflect pairs of day times that have a high chance of feeding with day times that have a low chance of feeding, e.g. feeding in the early evening with a feeding pause of 28 hours corresponds to the middle of the night, at which point feeding is highly unlikely.

An interesting question is why the activity at night and mid-day changes for female cheetah between the seasons (fig. 6). One hypothesis here is that female cheetah hunt at night if the environment allows it. During the wet season, cloud cover might reduce visibility at night and lead to a reduction in hunting behaviour. The increase of hunting during the middle of the day might then be a way to compensate for the reduction in night hunting. A point against the hypothesis is that male cheetah seem unaffected. But this might be due to gender differences, e.g. male cheetah might be more risk taking. Males are slightly larger than females and, in this specific case, M1 was in a coalition with another male so therefore not as vulnerable [22].

As a final word, we want to emphasize that, while in this paper we applied our method to data from cheetah, the approach is neither restricted to cheetah nor to feeding behaviour. In principle, any animal and any behaviour can be studied with the method as long as both observations and sensor measurements are available.
